# Innovations in Extractive Phases for In-Tube Solid-Phase Microextraction Coupled to Miniaturized Liquid Chromatography: A Critical Review

**DOI:** 10.3390/molecules25102460

**Published:** 2020-05-25

**Authors:** Henry Daniel Ponce-Rodríguez, Jorge Verdú-Andrés, Rosa Herráez-Hernández, Pilar Campíns-Falcó

**Affiliations:** 1 MINTOTA Research Group, Departament de Química Analítica, Facultat de Química, Universitat de València, Dr. Moliner 50, Burjassot, 46100 València, Spain; Henrypon@alumni.uv.es (H.D.P.-R.); Jorge.Verdu@uv.es (J.V.-A.); pilar.campins@uv.es (P.C.-F.); 2 Departamento de Control Químico, Facultad de Química y Farmacia, Universidad Nacional Autónoma de Honduras, Ciudad Universitaria, Tegucigalpa 11101, Honduras

**Keywords:** sorbents, in-tube solid-phase microextraction, capillary liquid chromatography, nano liquid chromatography

## Abstract

Over the past years, a great effort has been devoted to the development of new sorbents that can be used to pack or to coat extractive capillaries for in-tube solid-phase microextraction (IT-SPME). Many of those efforts have been focused on the preparation of capillaries for miniaturized liquid chromatography (LC) due to the reduced availability of capillary columns with appropriate dimensions for this kind of system. Moreover, many of the extractive capillaries that have been used for IT-SPME so far are segments of open columns from the gas chromatography (GC) field, but the phase nature and dimensions are very limited. In particular, polar compounds barely interact with stationary GC phases. Capillary GC columns may also be unsuitable when highly selective extractions are needed. In this work, we provide an overview of the extractive capillaries that have been specifically developed for capillary LC (capLC) and nano LC (nanoLC) to enhance the overall performance of the IT-SPME, the chromatographic separation, and the detection. Different monolithic polymers, such as silica C_18_ and C_8_ polymers, molecularly imprinted polymers (MIPs), polymers functionalized with antibodies, and polymers reinforced with different types of carbon nanotubes, metal, and metal oxide nanoparticles (including magnetic nanoparticles), and restricted access materials (RAMs) will be presented and critically discussed.

## 1. Introduction

Miniaturization in liquid chromatography (LC) has been a constant during the past decades, and nowadays, capLC and nanoLC equipment is readily available under a variety of configurations [1]. The main advantages of reducing the particle size and column diameter are the decrease on the consumption of both mobile and stationary phases, and a lower dispersion of the analytes on the column, which results in enhanced efficiency and mass sensitivity. Additional advantages are the lower consumption of sample, and a better compatibility with mass spectrometry detectors [2,3].

Because of the reduced column dimensions and flow rates, the sample volumes that can be handled in miniaturized LC systems are very low, ranging from a few µL in capLC to nL in nanoLC. This can be an advantage if the amount of sample available is limited, as often happens in the forensic and biomedical fields. However, in most cases, it makes necessary the proper sample treatment in order to reach the required analyte detectability.

In parallel, during the past years, a variety of sample treatments have been developed, many of them in the miniaturized scale. In this respect, solid-phase microextraction (SPME) has played a predominant role. Since its introduction [4], SPME has undergone rapid development, and it is currently used under different formats and in combination with many analytical techniques. In SPME, the sorbent is most often used as a fiber coating, but other supports are also used, such as particles, stir bars, fabrics, or capillary tubes (in-tube SPME). Moreover, the intensive research in the synthesis of new materials that can serve as sorbents, such as nanoparticles (NPs), monoliths, metal organic frameworks, or ionic liquids, has been very beneficial for SPME.

Among the several microextraction techniques available, IT-SPME (or capillary microextraction) is an essential contribution to the implementation of miniaturized LC because it is compatible with the direct processing of sample volumes much larger than those that can be handled directly with capLC and, specially, with nanoLC chromatographs. Other alternatives proposed to improve the analyte responses in this kind of system include column focusing, and two-dimensional approaches based on the combination of a cartridge column, which is used for analyte concentration and cleanup, and a separative column [5,6].

In IT-SPME, a tube containing the extractive phase, typically a capillary column coated or packed with the sorbent, is used as a replacement of the injection loop of the chromatograph (in-valve IT-SPME). The target analytes are trapped as the sample is being loaded in the capillary, and sent to the separative column with the mobile phase when the valve is changed to the inject position. Unlike approaches that use column cartridges, IT-SPME may not be exhaustive if the volume the sample processed is high in comparison with the amount of extractive phase, but the required analyte detectability can be reached by increasing the sample volume passed through the capillary; water or the proper solvent can be passed through to the capillary after loading the sample, so that unwanted matrix compounds can be selectively flushed out. Although less used, another possibility is to aspirate and dispense the sample through the capillary several times until a sufficient amount of the analytes is trapped (draw/eject IT-SPME). The draw/eject approach has mainly been used when the sample volume available is low, for example, in biomedical applications, whereas in-valve IT-SPME is better suited for the analysis of larger sample volumes, being the modality of choice for the analysis of water samples. A detailed comparison of the instrumental requirements of both approaches, and their respective advantages and limitations can be found somewhere else [7,8,9,10,11].

In IT-SPME, the amount of analyte trapped in the extractive capillary and, therefore, the sensitivity attainable, is mainly determined by the length of the capillary and by the sorbent contained in it. In principle, for a given sample volume, the amount of analyte trapped on the sorbent can be increased by increasing the length of the extractive capillary, and applications can be found in the literature with capillary lengths up 60–80 cm in conventional-scale LC. However, in on-line IT-SPME, the dimensions of the capillary determine the plug of the solvent with the analytes transferred from the extractive capillary to the separative column, which indeed must be adequate to the dimensions of the chromatographic system. Moreover, at the low mobile phase flow rates of capLC and nanoLC, long capillaries cause an excessive delay in the time required for the target compounds to reach the detector (which in turn may result in excessive peak broadening). The dimensions of the other elements necessary to set up the extraction device (connectors, injection valve) must also be properly selected to prevent band broadening.

In early applications of IT-SPME, commercially available columns from the GC field were primarily used. For example, Gou and Pawliszyn first reported the coupling of IT-SPME combined to capLC for the extraction of some carbamates from water samples using a segment of a polyethylene glycol (PEG)-coated GC column [12]. IT-SPME was carried out under a draw/inject scheme, and on-column focusing was employed in order to achieve good efficiency. In such a way, the detection limits were lowered by factors of 24–62 with respect to those achieved with the equivalent IT-SPME conventional-scale LC method.

In the following years, other commercially available capillary GC columns were used in combination with capLC and nanoLC, with those coated with polydimethylsiloxane (PDMS) stationary phases being predominant. As demonstrated by Campíns-Falcó and coworkers, these phases proved to be very useful for the extraction of a wide variety of compounds from water matrices, mostly organic pollutants, such as triazinic [6] and organophosphorous herbicides [13], polycyclic aromatic hydrocarbons (PAHs) [14], sterols [15], phthalates [14,16], and their degradation products [17], or chloramines [18]. These coatings were also used to process the extracts obtained from other environmental matrices. For example, a TRB 5 capillary column (PDMS with 5% of diphenyl groups) was used for the isolation of phthalates from the extracts obtained after applying matrix-solid phase dispersion (MSPD) to bivalves [19] and coastal sediments [20], as well as for the extraction of unmetabolized dialkyl phthalates from urine samples [21]. A TRB-35 column (PDMS with 35% of diphenyl groups) was used to study Ag NPs in soils [22]. Other applications of PDMS phases in the environmental area include the analysis of chlorophyll a in aquatic systems [23], and the extraction of compounds containing carbonyl groups in the aqueous extracts obtained by treating the filters used to collect atmospheric particulate matter [24,25]. Successful examples in other areas include the extraction of some pharmaceutical compounds [26,27] and surfactants [28,29], as well as different applications in the forensic field [30,31].

The first studies with PDMS phases consistently showed that the effectiveness of the extraction was mainly related to the polarity of the analytes, as observed for different classes of pollutants. For example, extraction recoveries <10% were found for polar triazinic herbicides with TRB 5 columns [6]; even though an increment of the thickness coating had a beneficial effect on the extraction rates, little improvement was achieved for highly polar organophosphorous compounds [13]. For these reasons, major efforts have been undertaken to develop new sorbents for IT-SPME capable of providing a high extraction efficiency, particularly for highly polar compounds.

On the other hand, commercially available columns interact with a wide variety of compounds, which is an advantage in terms of applicability, but may lack the required selectivity for the direct analysis of complex matrices. The development of highly selective sorbents for IT-SPME is, therefore, a key factor to extend the real utility of miniaturized LC.

**Figure 1:**
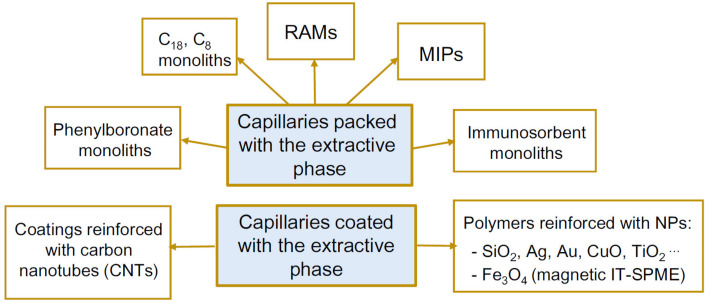
Summary of the extractive phases developed for IT-SPME coupled to capLC and nanoLC.

In recent years, different review articles have been published dealing with the advantages and limitations of IT-SPME with respect to other extraction and microextraction techniques, the configurations and extractive phases most commonly used, and relevant applications in different areas, but they were mainly focused on the conventional-scale LC [7,8,9,10,11]. The aim of the present review is to summarize the different sorbents that have been specifically developed for IT-SPME combined to miniaturized LC systems with the aim of enhancing the extraction efficiency and/or the selectivity of the extraction. The research articles included in this work were found in the ISI-Web of Science and Scopus databases (accessed May 2020) using the search topics “capillary liquid chromatography” and “in-tube solid-phase microextraction”, and “nano liquid chromatography” and “in-tube solid-phase microextraction”. According to the results of that search, the sorbents proposed, either as capillary packings or as coatings, are those depicted in Figure 1. In the next sections, the methods of preparation, extraction capabilities and relevant applications of those sorbents will be discussed. A summary of the articles included in this work can be seen in Table 1.

## 2. Capillaries Packed with Monoliths

The advantages of monolithic materials as extractive sorbents for sample preparation include their tunable properties and high permeability [59]. The specific advantages of monolithic material-packed capillaries over conventional microparticle-packed capillaries are a low-pressure drop (which facilitates sample loading), fast mass transfer kinetics, and high loading capacity. Compared with coated capillaries, monolithic-packed capillaries offer higher specific areas for interaction. Different monolithic polymers have been synthesized inside column capillaries, taking advantage of in situ polymerization reactions, and used for IT-SMPE in both capLC and nanoLC.

### 2.1. Octadecyl and Octyl Silica Monoliths

Monolithic-packed columns with an octadecylsilica (C_18_) phase were first proposed by Shintani and coworkers for IT-SPME coupled to capLC [32]. The polymer was prepared by in situ hydrolysis and polycondensation of tetramethoxysilane and poly(ethylene oxide) in an acetic acid media into a fused silica capillary tube (previously activated with a solution of sodium hydroxide); the monolithic formed was properly washed and treated with octadecyldimethyl-*N*,*N*-diethylaminosilane to form the C_18_ phase. Capillaries of 15 cm length and 0.2 mm i.d. with the synthetized phase were used to extract some pesticides. Despite using an in-valve configuration, the samples (0.1 µL) could be manually injected. Compared with the results obtained by extractive capillaries coated with a PDMS phase, the C_18_ monolithic phase led to higher extraction efficiencies.

A C_18_ monolithic column (15 cm × 0.2 mm i.d.) was used by Jia and coworkers in an in-valve configuration and coupled on-line to a capLC system [33]. The authors used a mobile phase containing tetrabutylammonium in order to form ionic pairs with the analytes. The so-called in-tube solid-phase ion-pair microextraction (SPIPME) method was applied to evaluate the concentration of flavins in *E. coli* cell extracts. A similar extractive phase was subsequently used for the extraction of fat-soluble vitamins and β-carotene in extracts obtained from corns [34].

An octyl (C_8_) functionalized hybrid silica monolithic column was used by Zheng and coworkers for the extraction of some PAHs [35]. The extractive phase was synthetized by a two-step acid/base-catalyzed hydrolysis/co-condensation of tetraethoxysilane (TEOS) and *n*-octyltriethoxysilane (C_8_-TEOS) inside the capillaries (15 cm × 0.25 mm i.d.). The size of through-pores, which had a strong effect on the extraction of PAHs, was adjusted by changing the ratio of TEOS to C_8_-TEOS in the polymerization mixture. The hybrid silica monolithic column allowed the injection of up to 1 mL of the samples without any loss in resolution. Hydrophobic interactions were responsible for a remarkable extraction capacity, and enhancement factors of 254–372 were found with respect to the untreated samples.

### 2.2. Phenylboronate Monoliths

Taking advantage of the reversible covalent reaction between boronic acids and small molecules containing cis-trans diol groups, Lin and coworkers developed a phenylboronate monolithic sorbent for the selective isolation of glycoproteins from unfractionated protein mixtures [36]. The sorbent was synthesized by introducing inside a 25 cm × 0.1 mm i.d. capillary a mixture of the monomer 4-vinylphenylboronic acid (VPBA), the crosslinker ethylene dimethacrylate (EDMA), diethylene glycol, and ethylene glycol as porogenic solvents, and azobisisobutyronitrile (AIBN) as the initiator. The resulting (VPBA-co-EDMA) polymer showed different affinities for glycoproteins and nonglycoprotein, which allowed its successful application for the specific capture of ovalbumin from egg white samples and their analysis by capLC.

A polymer of 3-acrylamidophenylboronic acid (AAPBA) and EDMA was proposed in [37]. The monolith was synthetized in 0.53 mm i.d. capillaries with a mixture of AAPBA, EDMA, methanol, PEG, and AIBN. The resulting monolith was successfully used to extract a mixture of nucleosides (cytidine, thymidine, adenosine, guanosine, and uridine) from urine samples (0.8 mL).

A boronate affinity capillary column was also used for purification/preconcentration of cis-diol nucleosides prior to their separation by nanoLC under reversed phase conditions in [38]. In this case, the extractive capillary (1 cm × 0.075 mm i.d.) was connected to a 7 cm length reversed phase monolithic column using a capillary electrophoresis system operating under pressurization mode.

### 2.3. Monolithic Molecularly Imprinted Polymers (MIPs)

Monolithic MIPs have attracted great interest in SPME because they combine easy preparation and improved selectivity. To date, MIPs have typically been used as packings of extraction cartridges for solid phase extraction (SPE), but they are also compatible with other forms of extractions, such as disks and fibers. They have also been synthetized into capillaries and used for IT-SPME, although in most cases coupled to conventional LC.

Szumski et al. synthetized monolithic MIPs inside fused-silica capillaries that were subsequently applied to the extraction, separation, and detection of aflatoxins by capLC [39]. A two-step polymerization process was used. First, a poly-(trimethylolpropanetrimethacrylate) core monolith was synthesized using UV photopolymerization. In a subsequent step, the obtained polymer was grafted with a mixture of methacrylic acid as the functional monomer, ethylene dimethacrylate as a cross-linking agent, 5,7-dimethoxycoumarin as an aflatoxin-mimicking template, toluene as a porogen, and AIBN. Once the synthesis and the rest of the experimental conditions were optimized, the capillaries (17 cm × 0.1 mm i.d.) were used for the extraction of aflatoxins B1, B2, G1, and G2 from aqueous solutions, with the volume of sample injected being 5 µL.

More recently, a monolithic MIP was synthetized by Bouvarel et al., and used for the selective extraction of cocaine and its main metabolite benzoylecgonine, prior to their analysis by nanoLC [40]. The polymer was synthetized inside fused-silica capillaries (previously activated with a solution of NaOH) using 3-(trimethoxysilyl)propyl methacrylate (MAPS) in ethanol for silanization, using a mixture of the template molecule (cocaine), methacrylic acid (functional monomer), trimethylolpropane trimethacrylate (cross-linker), and AIBN dissolved in the porogen (acetonitrile/isooctane). Segments of the obtained capillaries (5 cm × 0.1 mm i.d.) were connected to the nanoLC system via a six-port nano-valve. An additional nano-valve equipped with a 50-nL loop was used for injection of the samples. The proposed method was successfully applied to the analysis of cocaine in human plasma (previously treated with acetonitrile for protein precipitation).

### 2.4. Immunosorbent Monoliths

Considering the high specificity and affinity of the antigen–antibody interactions, immunosorbents have gained attention for the efficient and selective cleanup in the analysis of complex samples. Besides its inherent advantages, miniaturization is an attractive option when using this kind of sorbent due to the high cost of antibodies.

A hybrid organic-inorganic immunosorbent monolith was synthesized by Brothier and Pichon using a two-step process [41]. Activated fused silica capillaries (10 cm × 0.1 mm i.d.) were first filled with a mixture of hexadecyltrimethylammonium bromide (CTAB), TEOS, and 3-aminopropyltriethoxysilane (APTES) in a water-ethanol media. Once the polymer was formed and cleaned, monoclonal antibodies specific for the toxine microcystin-LR were grafted by pumping solutions of glutaraldehyde and the monoclonal antibodies. The capillaries were finally conditioned and coupled to a nanoLC system via a switching valve. An additional nanopump was used to deliver the solvent necessary to wash the capillary after sample loading (150 nL). The proposed method was successfully applied to the extraction of microcystin-LR from blue-green algae extracts. The same authors synthetized a DNA aptamer-based monolith by a similar procedure but using a 5′-amino-modified DNA aptamer with a C_12_ spacer arm specific for small molecules [42]. The extractive capillaries obtained (7 cm × 0.1 mm i.d.) were used in the analysis of ochratoxin A in beer samples by nanoLC.

A two-step procedure was also applied by Levernæs et al. for the synthesis of an immunosorbent with high affinity for the extraction proteotypic peptides from digested serum samples [43]. First, 15 cm × 0.18 mm i.d. capillaries were conditioned and silanized with MAPS in dimethylformamide (DMF). The monolith was subsequently formed inside the capillary using a mixture of EDMA and vinyl azlactone (VDM) in 1-propanol and 1,4-butandiol, in the presence of AIBN. The monoclonal anti-protein antibodies (mAb M18) were subsequently immobilized onto the EDMA-VDM polymer. The resulting capillaries were installed in a 10-port switching valve and connected to a nanoLC equipment. The proposed set-up was used for the determination of proteotypic peptides from digested serum samples.

## 3. Capillaries Packed with Restricted Access Materials (RAMs)

RAMs materials have been used for many decades for the selective extraction of a wide variety of small molecules from biological fluids. The utility of such materials for on-line microextraction coupled to miniaturized LC was demonstrated d by Santos-Neto et al. [44]. The authors synthetized a bovine serum albumin (BSA)-modified C_18_ extractive phase, which was used for the analysis of the antidepressant drug fluoxetine in human plasma. The capillaries (5 cm × 0.52 mm i.d.) were first packed with 0.45-µm C_18_ particles, and then successively flushed with methanol, phosphate buffer (pH 6.0), a solution of BSA, and a solution of glutaraldehyde. Finally, the capillaries were washed with a solution of sodium borohydride and water before use. The resulting capillaries were coupled to a capLC system, which showed a good tolerance to the injection of 1.5 µL of the samples. In a subsequent work, the BSA-C_18_ phase was compared with some commercial silica-based RAMs. According to the authors, the silica-based RAMs were found to be a better option in terms of reusability [45].

## 4. Coatings Reinforced with Carbon Nanotubes (CNTs)

Carbon-based nanomaterials, especially CNTs and graphene, have been extensively used as sorbents for (micro)extraction because of their high surface-to-volume ratio, and also because they can be obtained and functionalized easily. These materials can establish hydrogen bonding, stacking, and hydrophobic interactions with the analytes, thus providing suitable extraction efficiencies for a variety of organic compounds [60,61].

The possibility of increasing the extraction efficiency of PDMS phases by the incorporation of CNTs was tested for a variety of organic pollutants using TRB-5- and TRB-35-coated capillaries by Campíns-Falcó et al. [46,47]. The capillaries were functionalized with single-wall carbon nanotubes (SWCNTs) and multiwall carbon nanotubes (MWCNTs), previously carboxylated with a mixture of H_2_SO_4_:HNO_3_. The capillaries were first activated with a solution of NaOH, and then treated successively with APTES, glutaraldehyde, and solutions of the carboxylated CNTs (c-SWCNTs or c-MWCNTs) dispersed in a mixture of DMF and 1,3-dicyclohexylcarbodiimide; finally, the capillaries were flushed with water to eliminate the unbonded CNTs. The untreated TRB 5 and TRB35 capillaries and the functionalized capillaries were evaluated for different organic pollutants, nitrogen heterocyclic compounds, and PAHs using capLC. The affinity of most pollutants for the TRB-35 phases was higher than for the TRB 5 phases, whereas the functionalization of the coating did not increase the efficiency of the extraction for the most polar compounds tested (2-hydroxy terbutylazine and desethylterbutylazine). However, a significant increment on the extraction rate was obtained with the functionalized phases for pyriproxyfen, the least polar compound assayed. For the PAHs tested, the best results were obtained with the TRB 5 column functionalized with the c-SWCNTs phase. These results indicated that the presence of CNTs significantly increased the affinity of the coating for the analytes due to the additional π–π interactions established between the aromatic rings of the analytes and the NPs.

Similar results were observed for amphetamines after being derivatized with the fluorogenic reagent 9-fluorenylmethyl chloroformate [48]. The derivatives formed were subsequently analyzed by IT-SPME-capLC. In this case, a c-MWCNTs functionalized TRB 35 column provided the highest extraction efficiency for the target compounds. This synthetized coating was used to analyze amphetamines in oral fluid, using cotton swabs for direct sampling. Despite the complexity of the matrix, the performance of the IT-SPME was suitable for the determination of amphetamines in the oral fluids of abusers.

The employment of a TRB 5 capillary with carboxylated CNTs was used for the extraction of diclofenac by IT-SPME coupled on-line to nanoLC [49]. The presence of the CNTs enhanced significantly the extraction efficiency with respect to the unmodified TRB-5. In this case, the best results were obtained with c-SWCNTs.

## 5. Coatings Reinforced with SiO_2_ NPs

Silica NPs have long been used for sample treatment because they can interact with a variety of compounds through ion–dipole, dipole–dipole, and hydrogen bonding interactions [60]. In the context of miniaturized LC, SiO_2_ NPs were first used to reinforce a polymer synthetized from TEOS and triethoxymethylsilane (MTEOS) that served as a coating for IT-SPME coupled on-line to nanoLC by Campíns-Falcó and coworkers [50]. The capillaries (previously activated) were prepared by flushing through them a mixture of PEG, TEOS, MTEOS, a solution NH_4_OH in water, and variable amounts of SiO_2_ NPs. The images of the synthesized capillaries obtained by scanning electronic microscopy (SEM) revealed that the SiO_2_ NPs increased the porosity of the polymer. Segments of 15 cm × 0.075 mm i.d. of the synthetized capillaries allowed the direct introduction of up to 500 µL of the samples, which in turn resulted in improved analyte detectability with respect to that obtained by processing 4 mL of the samples with TRB 5, and TRB reinforced with CNTs capillaries and capLC. The new synthetized phase provided satisfactory recoveries of different triazines, including some degradation products, and good compatibility with different environmental matrices.

The extraction capabilities of TEOS-MTEOS phases were studied and compared with those obtained with commercially available GC columns of PDMS (TRB 5 and TRB 35), PEG (Carbowax), and nitroterephthalic acid-modified PEG (FFAP) [51]. The capillaries were coupled to a capLC instrument. Different herbicides with a wide range of polarities (log of octanol/water partition coefficients, log Kow ranging from −0.7 to 6.6) were selected as target compounds. The results of this study showed that with respect the unmodified TEOS-MTEOS polymer, the presence of SiO_2_ NPs substantially increased the extraction efficiency for all the tested compounds, but especially for the most polar ones. This was attributed to the additional interactions established between the free Si-OH groups on the surface of the NPs and the analytes. With respect the commercially available capillaries, the SiO_2_ NPs-modified coating provided the best extraction efficiencies for most of the tested compounds, although the nitroterephthalic acid-modified PEG coating showed higher affinity for compounds with certain functional groups (nitro-, carbonyl-). These findings confirmed that the interaction of the SiO_2_ NPs-modified TEOS-MTEOS coating was the result of the combination of hydrophobic interactions with the polymeric network, and hydrogen bonding and dipole–dipole interactions with free –OH groups of the SiO_2_ NPs. The capillaries coated with SiO_2_ NPs functionalized TEOS-MTEOS (30 cm × 0.075 mm i.d.) were applied to analyze the herbicides tested in different water samples, as well as in the extracts obtained from soil samples.

## 6. Phases for Magnetic IT-SPME

Magnetic NPs were first introduced by Campíns-Falcó et al. for IT-SPME as an attempt to increase the absolute extraction efficiencies of in-valve IT-SPME, which led to the so-called magnetic IT-SPME [52]. For this purpose, a magnetic hybrid extractive phase was formed by immobilizing Fe_3_O_4_ NPs supported on silica on the internal surface of fused silica capillaries. The coating was prepared by flushing through the activated capillaries a mixture containing the magnetic NPs in CTAB, PEG, urea, and TEOS. After the appropriate thermal treatment, a highly porous coating was obtained, in which the magnetic NPs were embedded. The extractive capillary was then placed inside a magnetic coil that allowed the application of a magnetic field, and installed in the injection valve of a capLC chromatograph. When applying a magnetic field, the NPs become magnetized, creating around them regions with different magnetic field gradients. When the working solutions are flushed through the capillary, diamagnetic analytes tend to be trapped and concentrated in the regions in which the field gradient is minimal. Next, the polarity of the applied magnetic field is reversed. As a result, the concentrated analytes are released and transferred to the analytical column with the mobile phase, as in ordinary IT-SPME. The utility of this methodology was demonstrated by processing some pharmaceutical compounds, namely acetylsalicylic acid, acetaminophen, atenolol, diclofenac, and ibuprofen. Magnetic IT-SPME provided extraction recoveries for the analytes within the 70%–100% range, whereas in the absence of a magnetic field, values of 10%–30% were obtained.

In subsequent studies, magnetic IT-SPME-capLC was successfully applied to the analysis of organophosphorous compounds in wastewater samples [53], and triazinic compounds in river water [54].

## 7. Coatings Reinforced with Metal and Metal Oxide NPs

Extractive capillaries coated with SiO_2_/PEG were reinforced with Fe_3_O_4_ NPs and tested for nanoLC by the Campíns-Falcó’s group in [49]. The target analyte was the emerging pollutant diclofenac. The coatings were prepared by introducing inside fused silica capillaries a mixture of PEG and urea in acetic acid, and dispersions of Fe_3_O_4_-CTAB NPs of different geometries; TEOS was added to form the SiO_2_-PEG matrix in which the Fe_3_O_4_-CTAB micelles were embedded. After the proper thermic treatment, a highly porous net was obtained, which showed a high affinity for diclofenac. The extraction efficiency was compared with that provided with other PDMS-based capillaries, some of them functionalized with CNTs. A 12 cm × 0.1 mm i.d. capillary column based on SiO_2_/PEG-supported Fe_3_O_4_ NPs was selected as a compromise between the enrichment factor, analysis time, and peak shape. It is remarkable that the capillary could be refilled up to six times with 8-µL aliquots of the sample before the desorption stage in order to improve the analyte detectability.

A TEOS-MTEOS polymer was doped with TiO_2_ NPs using a procedure similar to that described for the SiO_2_-TEOS-MTEOS phase, and used for IT-SPME coupled to both capLC and nanoLC in [55]. In the capLC system, the extraction efficiencies of the TiO_2_ NPs-doped phase were compared with those obtained with commercially available columns, namely TRB 35, FFAP, and polystyrene-divinylbenzene (PS-DBV), as well as with those obtained with SiO_2_ NPs-reinforced TEOS-MTEOS. For the nanoLC system, only the comparison between the TiO_2_ NPs and SiO_2_ NPs-doped TEOS-MTEOS phases was possible. The artificial sweetener saccharine and the PAHs naphthalene and fluoranthene were used as target compounds for capLC. It was observed that for the PAHs, the PS-DVB extractive capillaries provided the highest extraction efficiency, which was partially due to the higher thickness of the column coating. Despite the lack of polar functional groups in these compounds, the SiO_2_ NPs-doped coating led to better results than those obtained with the other commercial capillaries, which confirmed that the TEOS-MTEOS polymer was very efficient in extracting the analytes by hydrophobic interactions. For saccharine, also with an aromatic ring in its structure, the presence of the amino and sulfonyl groups led to the highest responses with the doped TEOS-MTEOS phases, although both the SiO_2_ and TiO_2_-doped phases provided similar results. In the studies with nanoLC, different types of herbicides were tested. In this case, significant differences in the extraction efficiencies were observed between the TiO_2_ and SiO_2_-doped coatings. The extraction efficiencies were generally better in the presence of TiO_2_ NPs, particularly for compounds with electronegative atoms in contiguous positions, most probably due to the bidentate-like interactions between the analytes and the NPs. On the other hand, SiO_2_ NPs-modified TEOS-MTEOS coatings were a better option for compounds having nitro groups. In many cases, the results obtained for compounds with similar K_ow_ coefficients were rather different, which proved that the extraction selectivity of the polymer could be changed by changing the NPs.

This possibility was further investigated using different metal and metal oxide NPs, namely Ag and Au NPs of different sizes and cappings, and a variety of metal oxide NPs (TiO_2_, ZrO_2_, Al_2_O_3_, Fe_2_O_3_, ZnO, and CuO NPs) [56]. A TEOS-MTEOS polymer modified with SiO_2_ NPs was also included for comparison purposes. The capillaries were tested for a variety of polar triazinic herbicides with log K_ow_ as low as −0.7, and the amino acids tryptophan (log K_ow_ = −1.06) and tyrosine (log K_ow_ = −2.26) The effect of the presence of NPs was evaluated by comparing the peak areas with respect to those obtained for the unmodified TEOS-MTEOS polymer. The best efficiencies for the most polar analytes (log K_ow_ ≤ 2.3) were observed with the CuO NPs, whereas the TiO_2_-doped phase gave the best responses for the least polar compounds. Based on those results, TEOS-MTEOS capillaries were synthetized using mixtures of CuO and TiO_2_ NPs, which increased the responses with respect to the unmodified polymer by factors up to 17.5. The serial coupling of two capillaries coated with modified with CuO and TiO_2_ NPs, respectively, and connected by means of a 10-port switching valve led to increments of the responses by factors up to 30 with respect the unmodified polymer. The reliability of the two-coupled capillaries approach was illustrated through the analysis of different river and sea water samples. Because of the high sensitivity attainable and the minimum sample manipulation involved, this approach is clearly advantageous in the analysis of species of limited stability, as it minimizes the impact of sample preparation on analytical results [57].

An additional advantage of the CuO TEOS-MTEOS phase is that it can be used in hydrophilic interaction chromatography (HILIC), which is the separation mode recommended for highly polar compounds. The utility of this bimodal extractive phase was demonstrated by determining the amino acids tyrosine and tryptophan under HILIC conditions, as a means of estimating the concentration of ovalbumin in wastewater samples [56].

## 8. Discussion and Future Trends

In the past years, a number of capillaries with sorbents of various chemical compositions have been proposed for complementing GC columns in order to improve the performance of IT-SPME coupled to capLC and nanoLC (Figure 1). Unlike the sorbent-packed capillaries used in conventional-scale IT-SPME, the new synthetized materials were prepared by in situ polymerization reactions taking advantage of the sol-gel reaction methodology. Interestingly, most of those phases have been specifically developed for nanoLC despite the fact that the number of IT-SPME-capLC methods is comparatively greater. This is the case of immunosorbents and MIPs, which have been primarily used for IT-SPME-nanoLC. This can be explained by different factors: (*i*) The necessity of improving the analyte detectability derived from low sample volumes that can be injected in nanoLC compared to capLC, (*ii*) the limited availability of commercial columns in dimensions adequate for nanoLC, and (*iii*) the increasing use of nanoLC in the biomedical field.

The intended benefits of the new proposals can be summarized in two main goals (see Table 2), the enhancement of the extraction efficiency though the development of phases with greater affinity for the analytes and/or greater active surface for interaction, and the synthesis of phases with the appropriate geometry or specific groups for interaction with the compound or compounds of interest.

Regarding the enhancement of the extraction efficiency, the early works were focused on the synthesis of the monolith version of traditional SPE sorbents (C_18_ or C_8_). However, the functionalization of commercially available capillaries with CNTs and the development of new polymeric coatings, many of them also reinforced with NPs, are currently the most used options. The advantages of nanostructured coatings are the remarkable increment of the active sites for interaction, and the possibility of tuning the extractive phase by using different NPs while maintaining favorable mass transfer rates. In these sorbents, it is essential to select the proper NPs in order to maximize their benefits. For example, CNTs are a good option for the extraction of aromatic compounds, whereas polymers doped with SiO_2_, CuO, or TiO_2_ NPs are a better option for the extraction of polar compounds with functional groups capable of interacting with the -OH groups of the NPs, as demonstrated with a variety of TEOS-MTEOS-doped phases. Since the polymer keeps its hydrophobic character, the extraction is the result of the combination of hydrophobic, ion–dipole, dipole–dipole, and hydrogen bonding interactions. In addition, at least two types of NPs with complementary selectivity can be combined in the extractive phase, so that it can be used for the extraction of compounds with a wide range of polarities. Another advantage of this kind of phase is that it maintains the extractive capabilities under different chromatographic conditions, such as reversed phase and HILIC conditions, as demonstrated with CuO NPs-doped TEOS-MTEOS. From another perspective, sorbents with magnetic NPs have been used to enhance the extraction efficiency by the magnetic IT-SPME approach. Monoliths capable of establishing combined interactions with the analytes can also be very useful. For example, a methacrylate polymer modified with the ionic liquid 1-butyl-3-vinylimidazoliumbromide was recently proposed for the enrichment of glycopeptide antibiotics in a cap LC system with amperometric detection [58]. According to the authors, the high extraction efficiency of the synthetized phase was the result of a mixed-mode mechanism involving hydrophilic, electrostatic, π–π interactions, and multipoint hydrogen bonding, due to the presence of the ionic liquid and a polar monomer (hydroxyethyl methacrylate) into the monolith. The potential of this kind of sorbents has not been sufficiently explored yet.

Regarding the phases aimed at improving the selectivity, RAMs, phenylboronate polymers developed for compounds with cis-trans groups, and monolithic MIPs for specific compounds have been synthetized. Of particular interest are the extractive capillaries packed with immunosorbents not only because of their specificity but also because due to the small dimensions of the extractive capillaries, the costs of the analysis are drastically reduced with respect to the equivalent normal-scale SPE-based methods.

**Figure 2:**
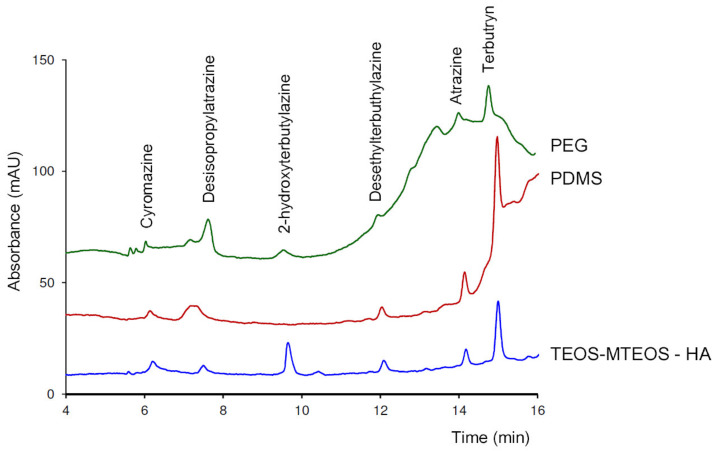
Comparison of the chromatograms obtained for some herbicides with a TEOS-MTEOS capillary modified with humic acid (HA), and with PDMS-based (TRB-5) and PEG-based (Wax Plus) commercial GC columns.

In the next years, progress can also be expected through the incorporation of other materials from the SPE technique. In this respect, a key aspect is the sustainability and costs of the sorbents. For example, there is an increasing interest in the development of sorbents based on humic acids because these naturally occurring substances are widely available, low cost, and unharmful [62], although no proposals for IT-SPME have been reported so far. Our research group has recently prepared a TEOS-MTEOS phase modified with humic acid (HA). In Figure 2, the chromatograms obtained by capLC for different herbicides with the TEOS-MTEOS-HA phase, and those achieved with segments of two commercial GC columns of the same length are compared.

As it can be seen in Figure 2, the new TEOS-MTEOS–HA phase not only increased the extraction yields for some of the analytes but also improved the overall chromatographic profile. New materials for SPE and SPME, such as metallic organic frameworks (MOFs), could also be good candidates for IT-SPME coupled to miniaturized LC.

## Figures and Tables

**Table 1:** Summary of the applications of IT-SPME with extractive phases developed for miniaturized LC.

Extractive Phase	Analytes	Sample Matrix	Column for Separation/Detection	Extraction Performance	Ref.
C_18_ monoliths	Pesticides	-	C_18_ (150 mm × 0.3 mm i.d., 3 µm)/UV–Vis	-	[32]
	Flavins	*E. coli* cell extracts	C_18_ monolithic (250 mm × 0.2 mm i.d.)/UV	Enhancement factors in peak heights relative to conventional injection of 110	[33]
	Fat-soluble vitamins and β-carotene	Corn extracts	C_18_ monolithic (270 mm × 0.1 mm i.d.)/UV	Enhancement factors in peak heights relative to conventional injection of 13–724	[34]
C_8_ monolith	PAHs	-	C_18_ (250 mm × 0.1 100 mm i.d., 3 µm)/UV-Vis	Enhancement factors on analytical responses of 254–372	[35]
4-phenylboronate affinity monolith	Glycoproteins	Egg white sample	-/UV	-	[36]
3-acrylamidophenyl boronate affinity monoliths	Nucleosides	Urine	Mehtacrylate based monolith (30 cm × 0.1 mm i.d.)/UV	-	[37]
	Nucleosides	-	RP-capillary column (7 cm-length)/UV (DAD)	Recoveries in urine, 86.5–106.8%	[38]
Methacrylate imprinted polymer	Aflatoxines B1, G1 B2 and G2	Water	Silica-based cholesterol (300 mm × 0.18 mm i.d.)/Laser induced fluorescence	-	[39]
Methacrylate imprinted monolith	Cocaine and benzoylecgonine	Plasma and saliva	C_18_ (150 x 0.075 mm i.d., 3 µm)/UV	Recoveries in plasma and saliva of 88.6–100.4%	[40]
Monolith with antibodies specific for microcystin-LR	Microcystin-LR	Cyano -bacteria cultures	C_18_ (150 mm × 0.1 mm i.d.)/UV (DAD)	Recovery > 70% in pure water	[41]
Monolithic with DNA aptamer	Ochratoxin A	Beer	C_18_ (150 mm × 0.1 mm i.d.)/Laser induced fluorescence	Recovery > 80% in standards	[42]
Monolith with anti-protein antibodies	Peptides	Digested serum	C_18_ (150 mm × 0.075 mm i.d., 3 μm)/MS/MS	Recovery of 110%	[43]
BSA modified C_18_ RAM	Fluoxetine	Plasma	C_18_ (100 mm × 0.52 mm i.d., 3 µm)/UV-vis	-	[44]
Different C_18_ and BSA modified C18 RAMs	Antidepressant and antihelmintic drugs	Plasma and urine	Capillary columns of variable length and i.d., packed with C18 120 Å pore, 5 µm particles/MS/MS	-	[45]
PDMS phase and PDMS doped with CNTs	Different pollutants and PAHs	Water	C_18_ (150 mm × 0.2 mm i.d., 0.5 μm)/UV(DAD)	CNTs increased the responses by factors up to 6.3	[46]
	Triazines	Sea and transition waters	C_18_ (150 mm × 0.2 mm i.d., 0.5 μm)/UV(DAD)	CNTs increased the responses only for the least polar analytes	[47]
	Amphetamines	Oral fluid	C_18_ (35 mm x 0.5 mm i.d., 3.5 µm)/Fluorescence	c-SWCNT increased the responses by factors of 2.9–3.3	[48]
PDMS phases doped with CNTs and Fe_3_O_4_ NPs; SiO_2_/PEG doped with Fe_3_O_4_ NPs	Diclofenac	Tablets and river waters	C_18_ (150 mm × 0.5 mm i.d., 3.5 µm)/UV(DAD)	Best sensitivity achieved with a SiO_2_/PEG supported Fe_3_O_4_ NPs phase	[49]
PDMS phases doped with CNTs and TEOS-MTEOS doped with SiO_2_ NPs	Polar triazines	Waters and recovered struvite	CapLC: monolithic C_18_ (150 × 0.2 mm i. d.) and C_18_ (150 mm × 0.5 mm i.d., 5 µm)/UV-Vis NanoLC: C_18_ (50 mm × 0.075 mm i.d., 3.5 µm)/UV(DAD)	Best analyte detectability achieved with the SiO_2_ NPs doped TEOS-MTEOS phase and nanoLC	[50]
TEOS-MTEOS doped with SiO_2_ NPs	Herbicides	Sea and transition waters; soil extracts	C_18_ (150 mm × 0.5 mm i.d., 5 µm)/UV(DAD)	Enrichment factors compared to commercial PDMS phases up to 5.1	[51]
Fe_3_O_4_ NPs supported on silica for magnetic IT-SPME	Pharmaceutical compounds	Water	C_18_ (150 mm × 0.5 mm, i.d. 3.5 μm)/UV(DAD)	Recoveries of 70–100%	[52]
	Organophosphorous compounds	Waste water	C_18_ (35 mm × 0.5 mm, 5 µm)/UV (DAD)	Recoveries of 94−97%	[53]
	Triazines	River water	C_18_ (150 mm × 0.5 mm, i.d., 3.5 μm)/UV(DAD)	Recoveries of 99–110%	[54]
TEOS-MTEOS doped with TiO_2_ NPs and SiO_2_ NPs	PAHs, saccharine phenylurea and organophosphorous herbicides	River and ditch water, and soil extracts	CapLC: C_18_ (150 × 0.5 mm i.d., 5 µm) /fluorescence NanoLC: C_18_ (50 mm × 0.075 mm i.d., 3.5 µm)/UV(DAD)	Best results for the most polar analyes obtained with the TiO_2_ NPs	[55]
TEOS-MTEOS doped with different metal and metal oxide NPs	Highly polar triazines and amino acids	Sea and river water	C_18_(50 mm × 0.075 mm i.d., 3.5 μm) and HILIC (150 mm × 0.075 mm i.d., 5 μm)/UV(DAD)	Enhancement factors with respect the TEOS-MTEOS phase up to 30	[56, 57]
Monolith with the ionic liquid 1-butyl-3-vinylimidazoliumbromide	Glycopeptide antibiotics	Feed extracts	Cyano (200 × 0.05 mm i.d., 3 µm)/Amperometry	Recoveries of 80–120%	[58]

**Table 2:** Criteria for selecting the extractive phase for IT-SPME.

Overall Objective	Target Compounds	Recommended Option
Enhancement of the efficiency	Compounds of low and/or medium polarity	Capillaries packed with organic silica monoliths (C_18_, C_8_)
		Polymeric phases reinforced with CNTs
		Sorbents for magnetic IT-SPME
	Extraction of compounds of very different polarities	Polymeric phases reinforced with a mixture of NPs
	Extraction of highly polar compounds	Polymeric phases reinforced with specific NPs (CuO, TiO_2_)
Enhancement of the selectivity	Extraction of small compounds from biofluids	RAMs
	Compounds with cis-trans diol groups	Capillaries packed with phenylboronate monoliths
	Specific compounds	Capillaries packed with MIPs
	Specific compounds	Capillaries packed with immunosorbents
